# Association of the Apolipoprotein E polymorphism with migraine: a meta-analysis

**DOI:** 10.1186/s12883-015-0385-2

**Published:** 2015-08-12

**Authors:** Jiayin Miao, Feng Wang, Weihong Zheng, Xiaorong Zhuang

**Affiliations:** Department of Neurology, Affiliated Zhongshan Hospital of Xiamen University, 201 Hubinnan Road, Xiamen, 361004 China; Department of Electronic Science, Fujian Provincial Key Laboratory of Plasma and Magnetic Resonance, Xiamen University, Xiamen, 361005 China; College of Computer Engineering, Jimei University, Xiamen, 361021 China

## Abstract

**Background:**

Apolipoprotein E (ApoE) gene has been reported to be associated with migraine and tension-type headache (TTH), but the results are conflicting. This study aimed to evaluate the association of ApoE with migraine by a meta-analysis.

**Methods:**

MEDLINE, ISI Web of Knowledge, The Cochrane Central Register of Controlled Trials, and EMBASE databases were searched to identify eligible studies published in English from 2000 to 2014. Data were extracted using standardized forms. The association was assessed by relative risk (RR) with 95 % confidence intervals (CIs) using a fixed or random effects model.

**Results:**

Four studies, comprising 649 migraineurs, 229 TTH subjects and 975 controls, met all the criteria and were included in the meta-analysis. No significant difference was found comparing genotypic and allelic frequencies in the case of migraineurs versus controls and TTH subjects versus controls. Only when migraineurs and TTH subjects were considered as a whole group, ApoE4 was found to increase the relative risk of headache by 1.48 (95 % CI 1.16, 1.90; P = 0.002), compared to controls.

**Conclusions:**

ApoE ε4 allele is not associated with migraine susceptibility, but is positively related to headache (including migraine and TTH).

## Background

Migraine is a common neurovascular disorder, characterized by recurrent episodes of headache and the dysfunction of the autonomic nervous system, affecting 10–20 % of the population [1, 2]. Although the pathophysiology of migraine is largely unknown, a neurogenic hypothesis of migraine has been proposed [3]. In particular, the genes involved in regulation of the endothelin system are proved to be promising candidates in migraine susceptibility [4, 5].

Apolipoprotein E (ApoE) is a 299 amino acid protein, encoded by a gene located on chromosome 19cen-q13.2 [6, 7]. ApoE gene has three common alleles (ɛ2, ɛ3, ɛ4) that encode three isoforms (APOE2, APOE3, APOE4). Several studies have shown a relationship between APOE polymorphism and the expression of the cytokines involved in migraine and TTH [8, 9]. Moreover, several studies investigated the association between APOE single nucleotide polymorphism (SNP) and migraine, but the results are conflicting [10–13].

Therefore, to define further the disease risk associated with APOE polymorphism, in this study we performed a meta-analysis of all related studies that evaluated allelic and genotypic frequencies of ApoE polymorphism in migraine.

## Methods

### Selection of studies

Eligible studies were identified by searching on web-based databases (MEDLINE 2000 to 2014, ISI Web of Knowledge 2000 to 2014, The Cochrane Central Register of Controlled Trials 2000 to 2014, and EMBASE) with no language restrictions. Search terms were “apolipoprotein E”, “APOE”, “ɛ2”, “ɛ3”, “ɛ4”, “APOE2”, “APOE3”, “APOE4”, “migraine”, “headache” and “Polymorphism”. Potentially relevant studies were retrieved and reviewed by 2 reviewers. All case–control studies with extractable data were included. Included studies were published as full-length articles in peer-reviewed journals.

### Data extraction and quality assessment

Studies were included for meta-analysis if they met the following criteria: (1) the association between APOE and migraine was examined by using a population-based, case–control design; (2) migraine was diagnosed using an international standard; (3) genotype or allele frequencies were reported in both cases and controls; (4) the genotype frequencies in control groups were consistent with Hardy-Weinberg equilibrium.

### Statistical analysis

Heterogeneity across the eligible studies was tested using Q-test, and it was considered statistically significant when P < 0.1. Heterogeneity was also quantified with I^2^ test (I^2^ = (Q− df) / Q × 100 %. I^2^ values of above 25 %, 50 %, and 75 % were taken as indicators of mild, modest, and high heterogeneities, respectively. When the effects were assumed to be homogeneous (*P* >0.1, I^2^ < 50 %), the fixed-effects model was used; otherwise, the random-effects model was more appropriate. The meta-analyses were performed using Stata Version 12.0 software (Stata Corp., College Station, TX, USA).

## Results

### Study inclusion and characteristics

As a result of our literature search, a total of four published articles reported on the relationship between APOE SNP and migraine and met the inclusion criteria. Rainero et al. divided the subjects into three subgroups: migraine with aura; migraine without aura; and mixed headaches (migraine associated with tension-type headache) [10]. Stuart et al. separated migraineurs into MA and MO groups. The other two reports investigated a possible association of APOE polymorphism with migraine and TTH [11, 12].

### Results of meta-analyses

Total 649 migraineurs, 229 TTH subjects and 975 controls were genotyped for APOE polymorphism. In the four studies, the distribution of the genotypes in control groups was in Hardy–Weinberg equilibrium (P < 0.05).

**Fig. 1:**
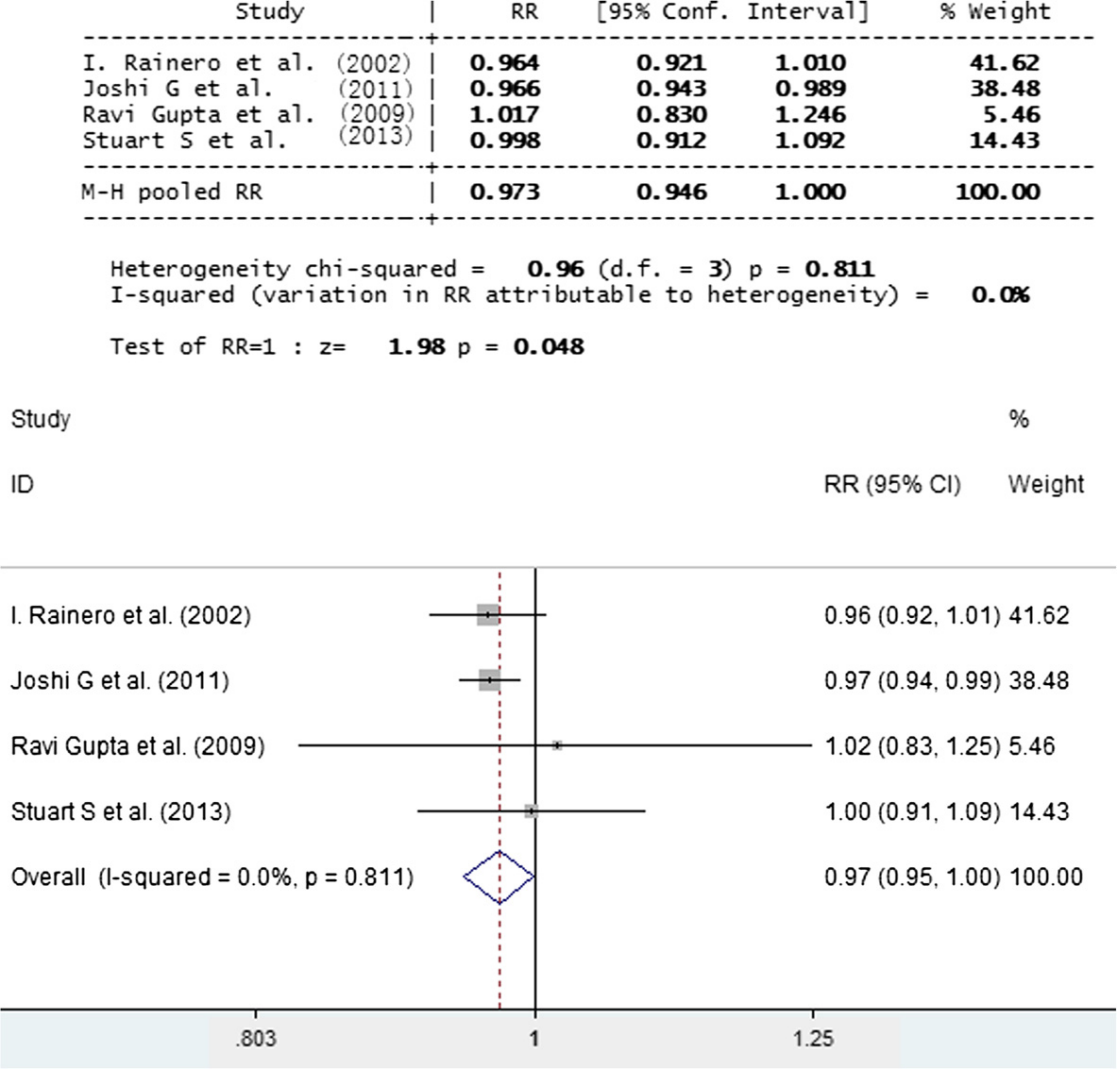
Forest plot of comparison: SNP APOE E3 of migraineurs and TTHs versus control. SNP APOE E3 was shown to have no significant association with migraineurs and TTH

**Fig. 2:**
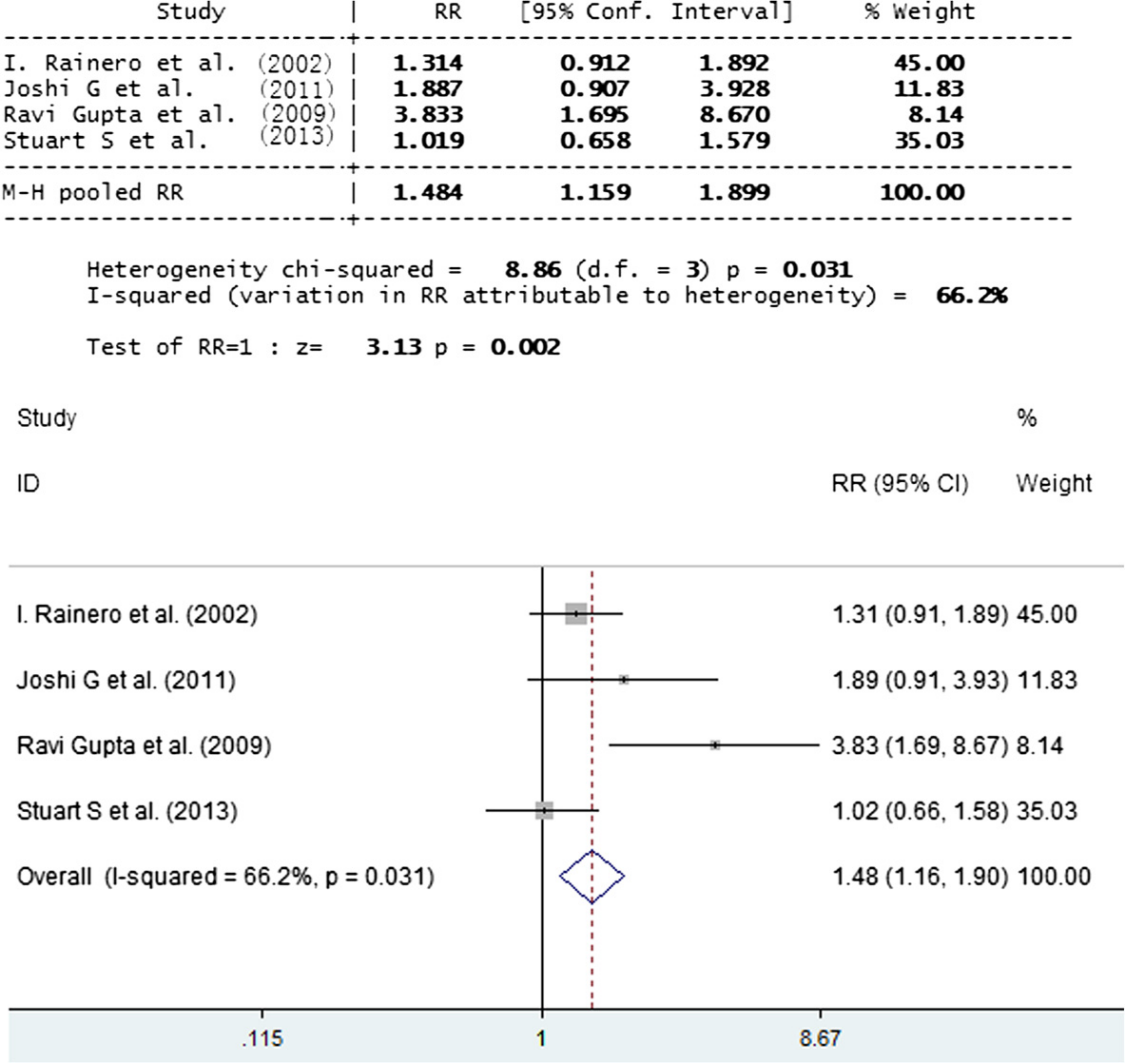
Forest plot of comparison: SNP APOE E4 of migraineurs and TTHs versus control. SNP APOE E4 was shown to have significant association with migraineurs and TTH

No significant difference was found between genotypic and allelic frequencies in the case of migraineurs versus controls and TTH subjects versus controls. Exclusively in the comparison between subjects with the allele E2 vs. E3 + E4, pooled RR with fixed effect was 1.49 (95 % CI 1.11, 2.01; P = 0.009, I^2^ = 74.1 %). This significance disappeared with random effect (RR1.82; 95 %CI 0.92, 3.61). While E4 gene increased the relative risk of headache by1.48 (95 % CI 1.16, 1.90; P = 0.002), they did not show any effect when migraineurs and TTH subjects were taken as a reference category (Figs. 1 and 2).

### Meta-analysis of subgroups

**Fig. 3:**
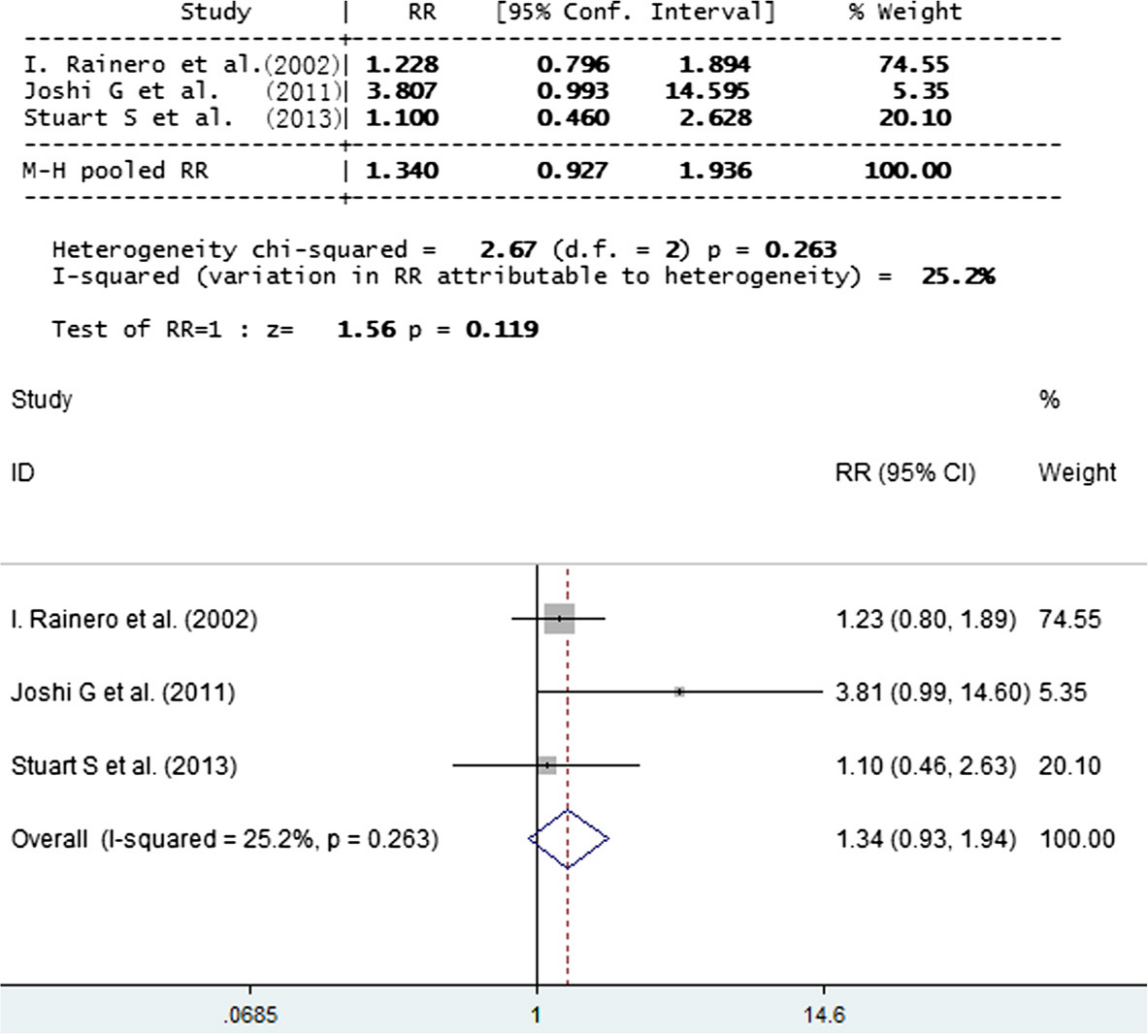
Forest plot of comparison: SNP APOE E2 of migraineurs of MO versus control. SNP APOE E2 was shown to have no significant association with migraineurs and TTH

**Fig. 4:**
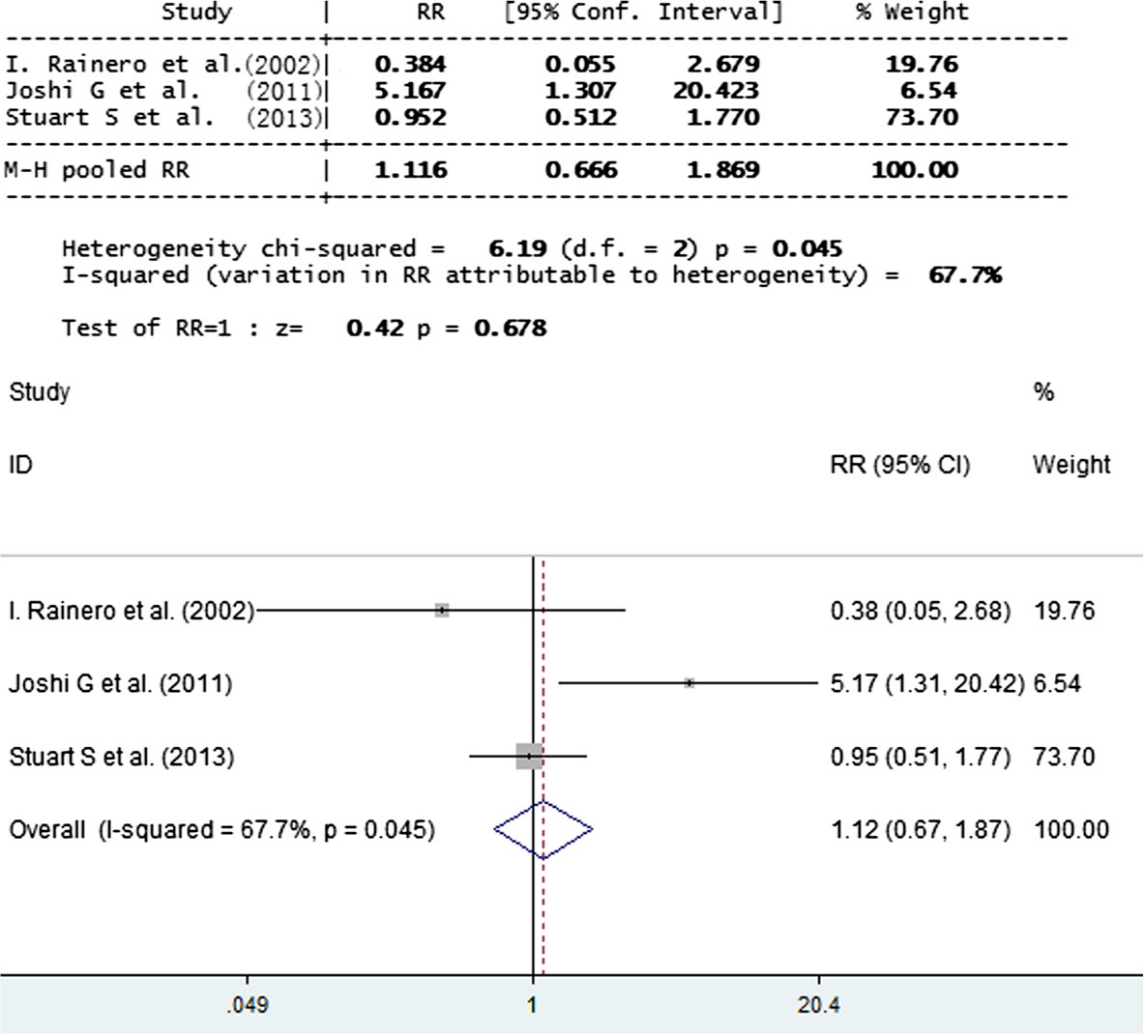
Forest plot of comparison: SNP APOE E2 of migraineurs of MA versus control. SNP APOE E2 was shown to have no significant association with migraineurs and TTH

To determine genetic heterogeneity of E2, MO and MA subgroups were also compared vs. controls. No significant difference between cases and controls was observed (Figs. 3 and 4).

## Discussion

The results of this meta-analysis suggest that there is no association between migraine and APOE polymorphism, even in subgroups analyses. However, when migraineurs and TTH subjects as a whole headache group were examined, a significant association was found between headache and E4 gene polymorphism. Migraineurs and TTH subjects carrying the allele E4, using fixed effects, showed a modest heterogeneity but significantly increased disease risk. The modest heterogeneity is the most likely explanation for the differences in ethnicity or in sample characteristics. Our data therefore partially confirm previous studies suggesting that this polymorphism represents a genetic risk factor for patients with migraine or TTH.

Nitric oxide (NO) plays an essential role in the pathogenesis of both migraine and TTH [14, 15]. Moreover, the production of NO is dependent on APOE HhaI polymorphism [16]. NO production is greater in APOE4 carriers with characteristically high levels of oxidative stress than in APOE3 carriers after closed head injury and stroke [17]. In addition, higher inflammation activity was associated with APOE ɛ4 gene compared to APOE ɛ3 gene [18]. Furthermore, APOE polymorphism influences the expression of the cytokines involved in migraine and TTH [8, 9, 19]. These results suggest that APOE4 may be associated with more serious tissue damage and higher disease susceptibility. Similarly, we found that APOE4 increased the relatively higher risk of headache compared to APOE3.

There are some limitations in our study. First, since migraine is a complex and heterogeneous disorder with a wide clinical spectrum, patients with different clinical phenotypes may not sufficiently capture this variability and may result in misclassification. Second, we only chose three genotypes in our study, despite various genotypes of APOE. Third, the lack of individual data has restricted further adjustments for the subgroup analysis for TTH. Fourth, the analysis was performed on a relatively small number of retrospective case–control studies, and the subjects included in these studies are also limited. Further population-based studies with larger sample size are necessary to confirm our conclusions.

## Conclusions

In summary, the evidence from the present meta-analysis showed that APOE4 is not associated with migraine or TTH, but is related to headache (including migraine and TTH), suggesting a nonspecific role for the risk of headache.
